# Assessing Exercise Limitation Using Cardiopulmonary Exercise Testing

**DOI:** 10.1155/2012/824091

**Published:** 2012-11-19

**Authors:** Michael K. Stickland, Scott J. Butcher, Darcy D. Marciniuk, Mohit Bhutani

**Affiliations:** ^1^Pulmonary Division, Department of Medicine, 8334B Aberhart Centre, University of Alberta, Edmonton, AB, Canada T6G 2B7; ^2^Centre for Lung Health, Covenant Health, Edmonton, AB, Canada; ^3^School of Physical Therapy, University of Saskatchewan, Saskatoon, SK, Canada; ^4^Division of Respiratory, Critical Care and Sleep Medicine and Airways Research Group, University of Saskatchewan, Saskatoon, SK, Canada

## Abstract

The cardiopulmonary exercise test (CPET) is an important physiological investigation that can aid clinicians in their evaluation of exercise intolerance and dyspnea. Maximal oxygen consumption (${\dot{V}}_{{\text{O}}_{\text{2max}}}$) is the gold-standard measure of aerobic fitness and is determined by the variables that define oxygen delivery in the Fick equation (${\dot{V}}_{{\text{O}}_{\text{2}}}$ = cardiac output × arterial-venous O_2_ content difference). In healthy subjects, of the variables involved in oxygen delivery, it is the limitations of the cardiovascular system that are most responsible for limiting exercise, as ventilation and gas exchange are sufficient to maintain arterial O_2_ content up to peak exercise. Patients with lung disease can develop a pulmonary limitation to exercise which can contribute to exercise intolerance and dyspnea. In these patients, ventilation may be insufficient for metabolic demand, as demonstrated by an inadequate breathing reserve, expiratory flow limitation, dynamic hyperinflation, and/or retention of arterial CO_2_. Lung disease patients can also develop gas exchange impairments with exercise as demonstrated by an increased alveolar-to-arterial O_2_ pressure difference. CPET testing data, when combined with other clinical/investigation studies, can provide the clinician with an objective method to evaluate cardiopulmonary physiology and determination of exercise intolerance.

## 1. Introduction

The cardiopulmonary exercise test (CPET) is an important physiological investigation that can aid clinicians in their diagnostic evaluation of exercise intolerance and dyspnea [1, 2]. Although cardiac and pulmonary etiologies are the most common causes for dyspnea and exercise intolerance [3, 4], neurological, metabolic, hematologic, endocrine, and psychiatric disorders can all contribute. The data gathered from a CPET can provide valuable information to differentiate between these causes [5], as progressive incremental exercise testing provides the most comprehensive and objective assessment of functional impairment and yields information about the metabolic, cardiovascular, and ventilatory responses to exercise. In addition to assisting in the diagnosis of dyspnea and exercise intolerance, CPETs can be used for a broad range of other applications such as determining disease severity, exercise prescription for rehabilitation, assessing the effectiveness of pharmacological agents, or in the assessment for lung transplant (see Table 1). 

Algorithms exist to help identify CPET patterns of known clinical diagnosis [6], and typical clinical responses have been detailed previously [1]. However, in order for clinicians to interpret CPET results, a thorough understanding of the cardiopulmonary responses to exercise is needed. The purpose of this paper is to provide the clinician with an overview of the physiological responses to exercise as well as the processes used to evaluate the mechanism(s) for exercise intolerance.

## 2. Cardiovascular Response to Exercise

Maximal oxygen consumption (${\dot{V}}_{{\text{O}}_{\text{2max}}}$) is a measure of the capacity for aerobic, and exercise is determined by the variables found in the Fick equation:

(1) $$\begin{array}{c} {\dot{V}}_{{\text{O}}_{\text{2}}}=Q\times \left(\text{Ca}{\text{O}}_{2}-\text{Cv}{\text{O}}_{2}\right), \end{array}$$

where  *Q*  is the cardiac output (the product of heart rate and stroke volume) and CaO_2_ and CvO_2_ are the O_2_ contents of arterial and mixed venous blood, respectively. From this equation, it is evident that the factors that influence  ${\dot{V}}_{{\text{O}}_{\text{2max}}}$  would include cardiac function, oxygen carrying capacity, and the ability of the tissues to extract oxygen.

In healthy subjects, of the variables involved in oxygen delivery, it is the limitations of the cardiovascular system that are most responsible for limiting ${\dot{V}}_{{\text{O}}_{\text{2max}}}$  [7]. Ventilation and gas exchange are usually sufficient to maintain arterial PO_2_ (PaO_2_), and therefore arterial saturation (SaO_2_) and CaO_2_ are also maintained up to maximal workload [8]. Numerous studies have shown that ${\dot{V}}_{{\text{O}}_{\text{2max}}}$ can be increased through exercise training [9, 10]. While peripheral adaptation occurs with training that will increase peripheral O_2_ extraction [11], the primary mechanism for training-induced improvements in ${\dot{V}}_{{\text{O}}_{\text{2max}}}$ is an increase in cardiac output secondary to an augmented stroke volume response to exercise [12]. Indeed, many studies have shown positive cardiac adaptation with exercise training [13–17]. The increased stroke volume response with exercise results in a reduced submaximal heart rate with exercise training; however, peak heart rate is generally unaffected by training [12]. Experimental studies have demonstrated that improvements in O_2_ delivery will positively affect ${\dot{V}}_{{\text{O}}_{\text{2max}}}$. As an example, Stray-Gundersen et al. showed that both peak cardiac output and ${\dot{V}}_{{\text{O}}_{\text{2max}}}$ could be increased by 20% in untrained dogs by performing pericardiectomy [18]. This effect is due to increased ventricular filling and thus an increased cardiac output. Conversely, a reduction in peak cardiac output will lead to a lower  ${\dot{V}}_{{\text{O}}_{\text{2max}}}$. This is highlighted by studies in normal humans showing beta blockade reduces ${\dot{V}}_{{\text{O}}_{\text{2max}}}$ by decreasing both maximal heart rate and stroke volume [19]. These examples from experimental studies demonstrate the close link between peak cardiac output and ${\dot{V}}_{{\text{O}}_{\text{2max}}}$ in health.

As ${\dot{V}}_{{\text{O}}_{2}}$ increases with incremental exercise, the variables in the Fick equation will eventually reach their upper limits, and as a result, a plateau of the ${\dot{V}}_{{\text{O}}_{2}}$ will occur. The plateau in oxygen consumption despite an increase in workload is defined as a person's ${\dot{V}}_{{\text{O}}_{\text{2max}}}$. However, many subjects, particularly clinical patients, do not demonstrate this plateau in ${\dot{V}}_{{\text{O}}_{2}}$ [20], for a variety of reasons which may include intolerable symptoms of breathing discomfort (dyspnea), muscular fatigue, chest pain, and so forth, [20, 21]. If a plateau is not seen, then the highest *V*_O_2__ achieved, termed the ${\dot{V}}_{{\text{O}}_{\text{2peak}}}$, is used as an estimate of ${\dot{V}}_{{\text{O}}_{\text{2max}}}$ [20, 22]. These values represent the maximal oxygen consumption and can be expressed in L/min or indexed by body weight and expressed in mL/min/kg [20]. Of note, the best adjustment for body size is not known and many estimations exist [20]. Various reference equations have been provided (see [1] for list) to evaluate ${\dot{V}}_{{\text{O}}_{\text{2max}}}$, and previous guidelines [1] define a ${\dot{V}}_{{\text{O}}_{\text{2max}}}<85\%$  of predicted as low and abnormal (see later section on evaluating  ${\dot{V}}_{{\text{O}}_{\text{2max}}}/{\dot{V}}_{{\text{O}}_{\text{2peak}}}$  for further discussion).

The limitation of the cardiovascular system is well accepted as being the point where healthy subjects reach their ${\dot{V}}_{{\text{O}}_{\text{2max}}}$  [23, 24]. Thus, if a subject reaches their maximum predicted heart rate (HR) for age (i.e., peak HR > 85% of predicted [1]), it would be reasonable to conclude based on the cardiac response that they have reached their ${\dot{V}}_{{\text{O}}_{\text{2max}}}$. However, this should not be used as a single determinant of  ${\dot{V}}_{{\text{O}}_{\text{2max}}}$, as there is considerable between-subject variability in maximal heart rate [25]. As well, clinical conditions and medications, especially beta blocker use, can affect the HR response to exercise [20–22]. Thus, in the setting of a reduced ${\dot{V}}_{{\text{O}}_{\text{2max}}}$, (i.e., <85% of predicted [1]), reaching maximal HR suggests maximal subject effort and that a cardiac limitation may exist; however, this must be confirmed by examining additional variables (see later section).

Oxygen pulse is the amount of oxygen consumed by the tissue per heart beat (i.e., ${\dot{V}}_{{\text{O}}_{2}}$/heart rate) [26]. By modifying the variables in the Fick equation, the O_2_ pulse is calculated as follows:

(2) $$\begin{array}{c} {\text{O}}_{2}\;\text{pulse}=\frac{{\dot{V}}_{{\text{O}}_{2}}}{\text{HR}}=\text{SV}\times \left(\text{Ca}{\text{O}}_{2}-\text{Cv}{\text{O}}_{2}\right). \end{array}$$

With O_2_ pulse, the assumption is that the *a* − *v*  O_2_ difference widens in a predictable manner, and therefore examination of the O_2_ pulse can provide information about the stroke volume response to exercise [26]. In the setting of a low ${\dot{V}}_{{\text{O}}_{\text{2max}}}$, a reduced O_2_ pulse would indicate a low stroke volume response to exercise. However, as O_2_ pulse is calculated using HR, the value is subject to the same assumptions regarding the HR response to exercise, and therefore the considerable between-subject variability in maximal heart rate [25] can translate to substantial variability in O_2_ pulse response to exercise.

In summary, the ${\dot{V}}_{{\text{O}}_{\text{2max}}}$ is determined by the variables that define oxygen delivery by the Fick equation. While anything that alters components of the Fick equation can alter ${\dot{V}}_{{\text{O}}_{\text{2max}}}$, studies in health have demonstrated that it is the cardiac output response and more specifically the stroke volume response to exercise that limit ${\dot{V}}_{{\text{O}}_{\text{2max}}}$, and thus in the normal healthy subject, ${\dot{V}}_{{\text{O}}_{\text{2max}}}$ is limited by the cardiovascular system.

## 3. Ventilatory Response to Exercise

As previously mentioned, ${\dot{V}}_{{\text{O}}_{2}}$ increases during exercise as governed, by the Fick equation. With increasing O_2_ consumption there is an increase in CO_2_ production  (${\dot{V}}_{{\text{CO}}_{\text{2}}}$). The relationship between PaCO_2_, ${\dot{V}}_{{\text{CO}}_{\text{2}}}$, and alveolar ventilation (${\dot{V}}_{A}$) is governed by the alveolar ventilation equation [27]:

(3) $$\begin{array}{c} \text{Pa}{\text{CO}}_{2}=\left(\frac{{\dot{V}}_{{\text{CO}}_{2}}}{{\dot{V}}_{A}}\right)\cdot \text{K}. \end{array}$$

PaCO_2_ is reported in mmHg (and assumed to be equal to alveolar PCO_2_), while both ${\dot{V}}_{{\text{CO}}_{2}}$ and ${\dot{V}}_{A}$ are reported in L/min [28]. ${\dot{V}}_{{\text{CO}}_{2}}$ is always given at 0°C, 760 mmHg, dry (STPD); ${\dot{V}}_{A}$ and PaCO_2_ are reported under body temperature, ambient pressure and saturated with water vapor (BTPS) [28]. The *K* is a conversion factor  [(273 + *t*) × 760/273], where *t* = body temperature (273 is 0°C converted to °Kelvin). *K* is used to adjust ${\dot{V}}_{{\text{CO}}_{2}}$ to body temperature and pressure and is equal to 863 mmHg at sea level and at normal body temperature of 37°C [27, 29]. As highlighted in (4) in the following section, ${\dot{V}}_{A}$ can be derived from ${\dot{V}}_{E}$ (minute ventilation) and ${\dot{V}}_{D}$ (physiologic dead space ventilation).

Assuming  *K*  does not change with exercise, (3) demonstrates that in order to maintain PaCO_2_ at normal resting values, ${\dot{V}}_{A}$ must increase with exercise because of the increased CO_2_ production. Thus in health, the normal response from rest to mild/moderate exercise is an increase in ventilation that is commensurate with metabolic demand (termed exercise *hyperpnea*), and therefore PaCO_2_ should be unchanged from rest to mild/moderate exercise. Practically, subjects often hyperventilate prior to exercise (or at low levels of exercise in the laboratory), and therefore it is common to see PaCO_2_ rise to a more normal value with mild/moderate exercise. Once past ventilatory threshold, ${\dot{V}}_{A}$ increases disproportionally relative to metabolic demand and PaCO_2_ drops below resting values (i.e., *hyperventilation*). PaCO_2_ typically falls to 30–35 mmHg at peak exercise, and a peak PaCO_2_ of 35–38 mmHg indicates a borderline effective alveolar hyperventilation, while a PaCO_2_ in excess of 38 mmHg suggests the absence of a compensatory hyperventilatory response [30]. Thus, PaCO_2_ values obtained with incremental exercise allow for the determination of the adequacy or appropriateness of ventilation during exercise.

End-tidal CO_2_ (PETCO_2_) can be used to estimate PaCO_2_. At rest PETCO_2_ is less than PaCO_2_ (and correspondingly end-tidal O_2_, PETO_2_ more than alveolar PO_2_, PAO_2_) due to dilution of gas from poorly perfused alveoli (i.e., dead space). Using end-tidal values to predict alveolar pressures has the potential of underestimating PaCO_2_; however, in the healthy lung at rest, dead space is extremely low, and PETCO_2_ is a good approximation of PaCO_2_ [28]. With exercise there is an increase in tidal volume (*V*_*T*_), ${\dot{V}}_{{\text{CO}}_{2}}$ and mixed venous CO_2_, such that the within-breath fluctuations of alveolar gas composition are greater [31]. With the rapid increase in alveolar volume on inspiration during exercise, end-inspiratory PCO_2_ is well below the mean alveolar PCO_2_, whereas during expiration, alveolar PCO_2_ increases toward mixed venous PCO_2_ more rapidly than at rest as the increased CO_2_ production of exercise is evolved into a lung volume becoming smaller as expiration continues [32]. The latter factor results in PETCO_2_ being higher than mean PaCO_2_ during exercise [33], and therefore PETCO_2_ has the potential to overestimate PaCO_2_ at peak exercise. In patients with lung disease who generally have a blunted tidal volume response to exercise, and a relatively low peak metabolic rate, the within-breath fluctuations of alveolar PCO_2_ are likely less than what would be seen in health. Rather, a larger issue in lung disease is the increased dead space ventilation and likely underestimation of PaCO_2_ using PETCO_2_. Jones et al. developed a prediction equation to calculate PaCO_2_ from PETCO_2_ during exercise [PaCO_2_ = 5.5 + (0.90 × PETCO_2_) − (0.0021 × tidal volume)] [32]; however, it is worth noting that this equation was developed with subjects exercising up to 50%  ${\dot{V}}_{{\text{O}}_{\text{2max}}}$. Further, it was suggested that the equation should not be used in patients with abnormal pulmonary function nor in children [32]. Thus, there are limitations with using PETCO_2_ as a prediction of PaCO_2_ that need to be considered when interpreting CPET data. Arterialized blood can also be used to predict PaCO_2_ with reasonable accuracy [34, 35] but is practically more difficult as compared to PETCO_2_.

## 4. Dead Space Ventilation

As shown in (4), total expired minute ventilation (${\dot{V}}_{E}$), measured at the mouth, consists of both alveolar ventilation (${\dot{V}}_{A}$) and physiologic dead space ventilation (${\dot{V}}_{D}$):

(4) $$\begin{array}{c} {\dot{V}}_{E}={\dot{V}}_{A}+{\dot{V}}_{D}. \end{array}$$

Alveolar ventilation is the amount of effective ventilation that participates in gas exchange. Physiological dead space is ventilation that does not participate in gas exchange and consists of anatomical dead space such as the conducting airways, as well as alveolar dead space which are unperfused alveoli. Physiological dead space can be calculated as a fraction of total ventilation using the Enghoff modification [36] of the Bohr [37] dead space equation:

(5) $$\begin{array}{c} \frac{{\dot{V}}_{D}}{{\dot{V}}_{E}}=\frac{\text{Pa}{\text{CO}}_{2}-\text{PE}{\text{CO}}_{2}}{\text{Pa}{\text{CO}}_{2}}, \end{array}$$

where PECO_2_ represented the mean PCO_2_ in the expired air. Examining this equation, dead space ventilation (i.e., ${\dot{V}}_{D}/{\dot{V}}_{E}$ ratio) would be zero if mean expired PCO_2_ was equal to arterial PCO_2_. Conversely, significant dead space results in expiration of gas that is more similar to inspired PCO_2_ (i.e., sections of the lung that did not participate in gas exchange and therefore have a  PCO_2_ ~ 0), which has the effect of diluting the expired air and reducing PECO_2_ relative to PaCO_2_. Of note, many metabolic carts typically calculate a dead space/tidal volume ratio (*V*_*D*_/*V*_*T*_ ratio, i.e., dead space per breath), using the same equation as listed in (5). However, these calculations are often based on a PaCO_2_ that is predicted from PETCO_2_, and therefore significant caution should be taken in interpreting *V*_*D*_/*V*_*T*_ values that are not derived using direct PaCO_2_ measurement.

## 5. Breathing Pattern Response to Exercise

The precise matching of alveolar ventilation with metabolic rate during exercise is achieved by increasing minute ventilation. This increase is accomplished by increases in both tidal volume and breathing frequency. The increased tidal volume slightly increases airway dead space, due to tethering effects of the lung parenchyma on airway lumen size. However, the relative tidal volume increase exceeds this effect, and the dead space to tidal volume ratio decreases during exercise from resting values of ~0.35 to ~0.20, translating into more efficient ventilation [1]. During low-to-moderate intensity exercise, both tidal volume and breathing frequency increase roughly in proportion to exercise intensity, whereas at higher intensities, tidal volume reaches a plateau and further increases in ventilation are accomplished by increases in breathing frequency alone [1].

Increases in breathing frequency are accomplished by reducing both the inspiratory (*T*_*I*_) and expiratory times (*T*_*E*_). However, the ratio of inspiratory time to total breath cycle duration (*T*_TOT_), the duty cycle (*T*_I_/*T*_TOT_), increases only slightly during exercise (~0.40 at rest to ~0.50 during high-intensity exercise) [38]. The increase in tidal volume is achieved by reducing the end-expiratory lung volume (EELV) below the functional residual capacity (achieved by activating expiratory muscles) and increasing the end-inspiratory lung volume (see later section on EELV determination) [38]. At lower exercise intensities, increases in ventilation are mostly achieved through tidal volume changes, rather than just increasing breathing frequency, which would increase dead space ventilation and compromise effective alveolar ventilation. To minimize the work of breathing during heavier exercise, tidal volume increases only to ~70% of the vital capacity [39], as above this lung volume, lung compliance decreases markedly and the respiratory pressure production required for a given change in volume is very large, leading to exaggerated respiratory discomfort (i.e., dyspnea) [40].

## 6. Ventilatory Efficiency

Ventilatory efficiency is typically evaluated by the ${\dot{V}}_{E}/{\dot{V}}_{\text{CO}2}$ responses to exercise, and as the term implies, it provides information about the effectiveness of minute ventilation for a given metabolic rate. Importantly, ventilatory efficiency has been shown to be decreased in several clinical conditions including chronic obstructive pulmonary disease (COPD), pulmonary arterial hypertension (PAH) [41, 42], and in heart failure [43]. In patients with PAH [42] and chronic heart failure [43], the ${\dot{V}}_{E}/{\dot{V}}_{{\text{CO}}_{2}}$ ratio is predictive of mortality. Importantly, when ${\dot{V}}_{E}/{\dot{V}}_{{\text{CO}}_{2}}$ is elevated it is important to understand the underlying physiological mechanism for the increased ${\dot{V}}_{E}$ relative to metabolic rate. As shown in (4), ${\dot{V}}_{E}$ would be elevated because of an increase in dead space and/or alveolar ventilation. In pulmonary arterial hypertension, the characteristic response is of pronounced hyperventilation at rest and with incremental exercise likely because of stimulation of receptors in the lung secondary to high vascular pressures [44]. In this condition, the enhanced ${\dot{V}}_{E}/{\dot{V}}_{{\text{CO}}_{2}}$  response to exercise is secondary to greater ${\dot{V}}_{A}$ as demonstrated by a low PaCO_2_ (or PETCO_2_) throughout exercise [41, 42]. Patients with chronic heart failure (CHF) also show an exaggerated ${\dot{V}}_{E}/{\dot{V}}_{{\text{CO}}_{2}}$ response to exercise [43]; however, PaCO_2_ can appear normal in these patients [45], indicating that the increased ${\dot{V}}_{E}/{\dot{V}}_{{\text{CO}}_{2}}$ is secondary to enhanced dead space ventilation. 

Lung diseases associated with airflow limitation and/or a loss of elastic recoil can lead to altered ventilation/perfusion (${\dot{V}}_{A}/\dot{Q}$) matching in the lung [46]. As a result of the reduction in ${\dot{V}}_{A}/Q$  matching, physiological dead space is increased, and therefore *V*_*D*_/*V*_*T*_ and ${\dot{V}}_{E}/{\dot{V}}_{{\text{CO}}_{2}}$ will be increased with incremental exercise as compared to controls [47]. In these patients ${\dot{V}}_{E}/{\dot{V}}_{{\text{CO}}_{2}}$ is exaggerated while PaCO_2_ is normal or perhaps even elevated, indicating that the increased ${\dot{V}}_{E}$ for a given metabolic rate is secondary to increased dead space. This reduction in ventilatory efficiency can further compromise exercise tolerance and potentiate dyspnea in patients with obstructive lung disease as their ventilatory reserve is already reduced, and therefore they have both an inability to increase ${\dot{V}}_{E}$ because of airflow limitation, plus a need to have a greater ${\dot{V}}_{E}$ for a given metabolic rate because of altered ${\dot{V}}_{A}/\dot{Q}$ matching and the associated increased dead space ventilation. These examples highlight how the ${\dot{V}}_{E}/{\dot{V}}_{{\text{CO}}_{2}}$ and PaCO_2_ responses to exercise can be used to differentiate between pathologies and mechanisms of dyspnea.

## 7. Ventilatory Reserve

Traditionally, ventilatory reserve has been evaluated by examining how closely the peak minute ventilation on a CPET (${\dot{V}}_{E\;\max}$) approaches the greatest volume of gas that can be breathed per minute by voluntary effort, termed the maximal voluntary ventilation (MVV). Previous guidelines state that breathing reserve [$\text{BR}=(\text{MVV}-{\dot{V}}_{E\;\max})/\text{MVV}\times 100$] should be >15% at peak exercise [1]. This method provides a general approximation of ventilatory capacity, with little analysis required. Ventilatory reserve depends on two main factors: ventilatory demand and ventilatory capacity [46, 48]. Ventilatory demand is dependent on metabolic demand, body weight, mode of testing, dead space ventilation as well as neuroregulatory and behavioral factors [48]. Ventilatory capacity is affected by mechanical factors such as airflow limitation and operating lung volumes, ventilatory muscle function, genetic endowment, aging, and disease [48]. Ventilatory capacity can also be affected by bronchoconstriction or bronchodilation [48]. Thus, a reduction in ventilatory reserve may be explained by increased ventilatory demand (such as during heavy exercise in an athlete or with inefficient ventilation) and/or reduced ventilatory capacity (typically due to airflow limitation).

Importantly, there are limitations to determining MVV which can affect determination of ventilatory reserve, and further, there are mechanical differences between voluntary hyperventilation at rest and exercise-induced hyperpnea. When performing an MVV at rest, subjects often hyperinflate, which can increase work of breathing relative to the same ventilation during exercise [46, 49–51]. In addition, MVV is subject to patient effort, and with poor effort the MVV can be low and the calculated ventilatory reserve falsely reduced. Because of the difficulties in measuring MVV, it is often predicted based on FEV_1_ (typically FEV_1_ multiplied by 35–40) [48, 52], and as with any prediction equation, there is variance around the accuracy of this prediction. Most importantly, using only the breathing reserve does not provide any information about the mechanism of ventilatory constraint (i.e., is there evidence of expiratory flow limitation or hyperinflation?) [46]. It is for these reasons that examining expiratory flow limitation and operating lung volumes has evolved as the preferred technique to examine a ventilatory limitation to exercise. 

## 8. Expiratory Flow Limitation

To evaluate the degree of ventilatory constraint during exercise, the degree of expiratory flow limitation (EFL) can be examined by plotting the exercise flow-volume loop relative to the maximal flow [46]. This relationship can provide information about the degree of expiratory flow limitation, operating lung volumes, as well as breathing strategies used with incremental exercise. The degree of EFL during exercise has been previously expressed as a percent of  *V*_*T*_  that meets or exceeds the expiratory boundary [48, 53, 54]. The presence of EFL promotes dynamic hyperinflation and intrinsic positive end-expiratory pressure with increased work of breathing, functional impairment of inspiratory muscle strength, increased sensations of dyspnea, and adverse effects on hemodynamics [55, 56]. When the degree of expiratory flow limitation becomes significant (>40–50% ${\dot{V}}_{T}$), EELV typically increases [48, 53, 57, 58]. 

Many of the modern metabolic carts allow for evaluation of EFL by plotting exercise tidal breathing within a maximal flow-volume loop. However, there is no clear consensus regarding the quantification of EFL. Johnson et al. [48] suggested an evaluation criteria regarding EFL and inspiratory capacity (IC); however, this had not been widely adopted clinically. Instead, most typically categorize EFL as an “all or none” criteria. Importantly, it is not unusual for a normal young (<35 yrs) subject of average fitness and no lung disease to have EFL of <25% of *V*_*T*_ at peak exercise [48, 49, 59, 60]. Thus, the clinical significance of some EFL occurring at or close to peak exercise is unclear. 

By definition, EFL requires the demonstration of an increase in transpulmonary pressure with no increase in expiratory flow [56]. As well reviewed recently by Calverley and Koulouris [56], the comparison of tidal breathing relative to the maximal flow volume loop has its limitations including (1) thoracic gas compression artifact; to reduce these errors volume should be measured using a body plethysmograph instead of the typical Pneumotach. (2) Incorrect alignment of the tidal breathing curve within the maximal flow-volume loop. (3) The previous volume and time history of a spontaneous tidal breath is different than the flow-volume curve derived from the maximum forced vital capacity; there is not a single maximum flow volume curve, but rather a family of curves which are dependent on the time course of the preceding forced vital capacity [56, 61–63]. (4) Mechanics and time-constant inequalities are different in tidal versus maximal flow-volume curves. (5) Exercise may cause bronchodilation/bronchoconstriction. (6) The technique requires good patient cooperation/effort. Guenette et al. [64] recently demonstrated that failure to account for gas compression and exercise-induced bronchodilation results in a significant overestimation of EFL. As a result of these limitations, the use of plotting tidal breathing relative to the maximal flow-volume loop to detect/quantify EFL has been questioned [56], although many of these potential limitations can be avoided or minimized with the use of standardized techniques. 

As an alternative, the negative expiratory pressure method has been advocated for the detection of EFL. As the name implies, with this technique a small negative pressure (i.e., suction of −3 to −5 cm H_2_O) is given during expiration [56]. This method is based on the principle that in the absence of EFL, an increase in the pressure gradient between the alveoli and the mouth would increase flow, whereas with EFL increasing the pressure gradient would not increase flow [56]. This technique has been used during exercise to demonstrate EFL in lung disease [65–67]; however, it does not allow for quantification of severity of EFL and has not been adopted during widespread clinical practice.

## 9. Inspiratory Capacity

With EFL, expiratory flow rates are independent of expiratory muscle effort and are determined by the static lung recoil pressure and the resistance of the airways upstream from the flow-limited segment [60, 68, 69]. In flow-limited patients, the mechanical time constant for lung emptying is increased in many alveolar units, but the expiratory time available is often insufficient to allow EELV to return to its original values, resulting in gas accumulation and retention (i.e., air trapping) [60]. As demonstrated by (3), the increased CO_2_ production with exercise necessitates an increase in ${\dot{V}}_{A}$ by increasing *V*_*T*_ and breathing frequency to maintain PaCO_2_. However, the increased tidal volume in combination with diminished expiratory time due to increased breathing frequency can cause dynamic hyperinflation in patients with EFL [60]. Thus, the main consequence of expiratory flow limitation during exercise is the development of dynamic hyperinflation (DH) [47, 60].

As reviewed recently by O'Donnell and Lavenziana [60], DH during exercise has several important consequences including (1) a sudden increase in elastic and threshold loads on the inspiratory muscles, leading to increased work and O_2_ cost of breathing. (2) Functional inspiratory muscle weakness by shortening the diaphragm muscle length. (3) Reducing the ability of *V*_*T*_ to expand appropriately with exercise, leading to a mechanical limitation of ventilation. (4) Hypoventilation and hypoxemia in more severe patients [70]. (5) Impairment in cardiac function. In COPD patients, ${\dot{V}}_{{\text{O}}_{2\text{peak}}}$ was strongly related to peak tidal volume (*r* = 0.68), which in turn was strongly related to IC at peak exercise (*r* = 0.79) [71]. These results indicate that DH blunts the tidal volume expansion with incremental exercise, which contributes to exercise intolerance/reduced ${\dot{V}}_{{\text{O}}_{2\text{peak}}}$. Consistent with the consequences of IC listed, the IC during exercise and the rate of change in IC with exercise (i.e., dynamic hyperinflation) are strong determinants of exertional dyspnea and exercise intolerance [71–73]. 

Dynamic hyperinflation in early exercise may be a compensatory mechanism to increase ${\dot{V}}_{E}$ with limited (or minimal) respiratory discomfort [74]; however, with increasing exercise a threshold is reached (around an inspiratory reserve volume of 0.5 L, or within 10% of total lung capacity), where *V*_*T*_ plateaus [60, 74]. At this point the breathing occurs at the least compliant portion of the respiratory system's pressure-volume curve; the diaphragm muscle fibers are maximally shortened, and dyspnea develops at an extremely accelerated rate because of the disparity between the inspiratory effort and tidal volume response [60, 74]. 

Recent work has shown that below this tidal volume inflection (or plateau), dyspnea increases linearly with workload; however once IC drops below a critical value, dyspnea increases abruptly and becomes the most frequently selected reason for exercise termination regardless of exercise protocol [75]. The rate of dynamic hyperinflation has been shown to be correlated with diffusion capacity (DLCO/${\dot{V}}_{A}$) [71]. Patients with lower DLCO would be expected to have a greater propensity to expiratory flow limitation because of reduced lung elastic recoil and airway tethering. Patients with a more emphysematous clinical profile (i.e., low DLCO) have been shown to have a greater rate of dynamic hyperinflation, less expansion of tidal volume, greater dyspnea, and lower ${\dot{V}}_{{\text{O}}_{2\text{peak}}}$ as compared to patients with similar airflow obstruction, but normal DLCO [71]. More recent work has shown that in COPD patients it may be the progressive erosion of resting IC with worsening airflow obstruction and hyperinflation that represents the true operating limits for tidal volume expansion from rest to exercise [76]. O'Donnell et al. [76] found that reductions in resting IC were associated with the development of an increasingly shallow, rapid breathing pattern and worsening dyspnea at progressively lower levels of ventilation during exercise. Importantly, regardless of the severity of airflow limitation, once *V*_*T*_ reaches the previously described threshold, there was a steep increase in dyspnea [76]. Other recent work has shown that it may not be the drop in IC but rather a critical reduction in inspiratory reserve volume that causes the plateau in *V*_*T*_ and marked increase in dyspnea [77]. These findings indicate that EFL contributes to DH, and once EELV has increased to a critical value and/or inspiratory reserve volume drops to a critical value, dyspnea is greatly potentiated, resulting in substantial exercise limitation.

Serial inspiratory capacity maneuvers are used during incremental exercise to evaluate EELV/IC progression with exercise. The use of IC to track EELV during exercise is based on the assumption that total lung capacity (TLC) does not change during exercise, and that reductions in IC represent changes in EELV (i.e., EELV = TLC − IC) [78, 79]. Inspiratory capacity is determined by the degree of hyperinflation, inspiratory muscle strength, and the extent of intrinsic mechanical loading on the inspiratory muscles [72]. The IC also provides information regarding the position of the tidal volume on the respiratory system's pressure-volume curve [72]. The lower the IC, the closer towards TLC the subject is breathing, which is the least compliant portion of the respiratory system's pressure-volume curve. Previous work has also shown that IC determination can be reliably obtained during exercise [72, 80]. When performing serial IC measurements with incremental exercise, a good effort is required to inspire up to TLC during each maneuver so as to ensure IC is not becoming falsely reduced because of inadequate inspiration. Esophageal pressure data confirms that peak esophageal pressure (an estimate of effort) does not change with repeated IC measurements, thereby indicating that serial ICs are valid with incremental exercise testing [72, 73, 80]. In addition to IC maneuvers, changes in EELV during exercise can also be tracked with newer methods such as optoelectronic plethysmography or respiratory inductance plethysmography [81, 82]; however, these techniques have not been adopted widely for clinical use.

## 10. Pulmonary Gas Exchange

Pulmonary gas exchange is typically evaluated by alveolar-arterial oxygen partial pressure difference (AaDO_2_ = PAO_2_ − PaO_2_). The stress of exercise on pulmonary gas exchange can be highlighted by the following two equations. For a hypothetical homogeneous lung with no ${\dot{V}}_{A}/\dot{Q}$ heterogeneity, the physiological definition of lung diffusion capacity for O_2_ (DLO_2_) is [28]:

(6) $$\begin{array}{c} \text{DL}{\text{O}}_{2}=\frac{{\dot{V}}_{{\text{O}}_{2}}}{\text{PA}{\text{O}}_{2}-\text{Pc}{\text{O}}_{2}}. \end{array}$$

PcO_2_ is the mean PO_2_ passing through the pulmonary capillaries, which cannot be measured and therefore is estimated by arterial blood sampling. Assuming PcO_2_ = PaO_2_ this equation can be rearranged to:

(7) $$\begin{array}{c} \text{AaD}{\text{O}}_{2}=\frac{{\dot{V}}_{{\text{O}}_{2}}}{\text{DL}{\text{O}}_{2}}. \end{array}$$

This physiological definition demonstrates that with the increased O_2_ consumption with exercise, the lung must increase its diffusive capacity in order to limit the increase in AaDO_2_ [28]. DLO_2_ increases with exercise as a result of capillary recruitment, as demonstrated by an increase in diffusion capacity with exercise [83–88]. From this equation it is intuitive as to how exercise may result in impaired gas exchange in patients with lung disease, resulting in decreased ${\dot{V}}_{{\text{O}}_{\text{2max}}}$ and/or increased dyspnea. Patients with a diffusion impairment at rest from thickening of the blood gas barrier, such as in interstitial lung disease, would be expected to show an increase in AaDO_2_ with exercise, while patients who have an inability to recruit pulmonary capillaries and therefore increase DLO_2_ because of capillary destruction (i.e., COPD) would also increase AaDO_2_ with exercise. Importantly, in addition to the impact on recruitment of diffusion capacity, lung disease can also result in greater ${\dot{V}}_{A}/\dot{Q}$ mismatch which can be exacerbated with exercise, resulting in further deterioration in gas exchange.

In health, most exercising humans show an increase in AaDO_2_ with incremental exercise which reaches its peak at ${\dot{V}}_{{\text{O}}_{\text{2max}}}$  [30, 89], but remains within normal limits (i.e., <35 mmHg) [1]. The AaDO_2_ appears greatest in endurance athletes, and in severe cases may cause hypoxemia [30, 89], which is somewhat counterintuitive as one would expect endurance athletes to have an excellent cardiopulmonary system. The increase in AaDO_2_ with exercise has been an area of physiological interest and is likely explained by a combination of ${\dot{V}}_{A}/\dot{Q}$ mismatch [90–92] and diffusion limitation secondarily to reduced red blood cell transit time or the development of interstitial non-clinical edema [90–93] and/or the recruitment of intrapulmonary arteriovenous shunts [94, 95]. Importantly, despite the attention given to pulmonary gas exchange in the research literature, exercise-induced arterial hypoxemia is uncommon in all but the most highly aerobic athletes. Thus, further clinical followup may be warranted in symptomatic non-athletic subjects who demonstrate an exaggerated AaDO_2_ (>35 mmHg) and/or decreased PaO_2_ with exercise.

As measurement of PaO_2_ requires arterial catheterization, most CPET studies are conducted by monitoring arterial saturation by pulse oximetry (SpO_2_). While SpO_2_ may be appropriate for monitoring, care should be taken when interpreting this data. Firstly, the standard error of estimate for SpO_2_ monitors is between 2% and 5% [96–98]. SpO_2_ monitors can also bias low when blood flow is reduced, such as what can occur with a finger oximeter while subjects are exercising vigorously on a cycle ergometer. Previous work suggests that an oximeter placed on the forehead provides the most accurate readings [97]. When using SpO_2_ to evaluate gas exchange during normoxic exercise, it is important to note that within the typical exercise range, SaO_2_ values are on the flat part of the oxygen hemoglobin dissociation curve, and within this range relatively small changes in SaO_2_ are associated with large differences in PaO_2_. Thus, even small uncertainties in SaO_2_ would have a big effect on estimated PaO_2_ [97]. SaO_2_ is also affected by the temperature and pH changes during exercise, and these alone can result in a SpO_2_ decrease of 4%-5% in the absence of any change in PaO_2_. Finally, should hypoxemia develop, it is not possible to determine if hypoxemia is secondary to an impairment in gas exchange (i.e., increased AaDO_2_) or significant hypoventilation with a corresponding drop in PAO_2_ and PaO_2_. Previous guidelines [1] define an SpO_2_ of 88% during exercise as significant hypoxemia; however, this value does not rule out the development of a significant gas exchange impairment, and therefore temperature-corrected arterial blood gas data should be used if careful gas exchange evaluation is needed. 

## 11. CPET Interpretation

The purpose of the previous sections was to highlight the physiological responses to exercise, and how decrements in cardiopulmonary physiology can lead to dyspnea and exercise intolerance. While a great deal of research has examined cardiopulmonary physiology and exercise, these findings still make it somewhat difficult to integrate all the data obtained in a CPET to provide a clear clinical interpretation of the mechanism(s) contributing to dyspnea/exercise intolerance in symptomatic individuals. Previous position statements have provided insight [1], and the purpose of this section is to provide guidelines to help clinicians evaluate CPET responses. It should be noted that the interpretation strategy described may not apply to all conditions and remains an evolving process. It is also important to appreciate that there are various contraindications to CPET (see Table 2).

## 12. Determination of Maximal Patient Effort

Prior to full interpretation of a CPET, determination of maximal patient effort is required. Previous guidelines [1] list the following as evidence of maximal patient effort. (1) The patient achieves predicted ${\dot{V}}_{{\text{O}}_{2\text{peak}}}$ and/or a plateau in ${\dot{V}}_{{\text{O}}_{2}}$ is observed. (2) Predicted maximal work rate is achieved. (3) Predicted maximal heart rate is achieved. (4) There is evidence of a ventilatory limitation; that is, peak exercise ventilation approaches or exceeds maximal ventilatory capacity. (5) A respiratory exchange ratio (RER, often called respiratory quotient (RQ)) greater than 1.15. (6) Patient exhaustion/Borg scale rating of 9-10 on a 10-point scale.

Importantly, because of the cardiovascular adaptations observed in athletes, these subjects often exceed predicted ${\dot{V}}_{{\text{O}}_{\text{2max}}}$  and predicted maximal work rate even during submaximal work, and therefore we would suggest that reaching predicted or ${\dot{V}}_{{\text{O}}_{\text{2max}}}$  or maximum work rate should not be evidence of a maximal effort. Based on this and new research detailed previously on EFL and changes in IC with exercise, we would suggest the following criteria for determination of maximal effort.

**Criteria for Maximal Effort**

1. RER ≥ 1.1.
2. HR > 90% predicted max.
3. Patient exhaustion/Borg scale > 9/10.
4. Was there a plateau in ${\dot{V}}_{{\text{O}}_{2}}$?
5. Was there evidence of a ventilatory limitation (breathing reserve <15% and/or significant EFL and/or decrease in IC)?

Importantly, there is no gold standard for evaluating maximal effort [1]. There is currently disagreement as to whether hypoxemia is evidence of a maximal effort. As hypoxemia can develop during submaximal exercise in some patients (e.g., interstitial lung disease), it has been suggested that this is not evidence of a maximal test [1], while others have indicated that hypoxemia is indeed confirmation of a maximal test [99]. 

With respect to the above-listed criteria, when more criteria are attained during a CPET, there would be more confidence that a maximal patient effort has been obtained. Notably, patients often have difficulty reaching a plateau in ${\dot{V}}_{{\text{O}}_{2}}$, and considering the between-subject variability in maximal heart rate [25], both criteria (2) and (4) are frequently not reached despite maximal effort. Further, while patients may achieve exhaustion with CPET testing (3), their Borg scale may be high, but not exceed a value of “9” on Borg scale as defined by previous guidelines [1]. It is also important to note that in the absence of respiratory disease, criteria (5) is rarely obtained. Conversely, in the presence of a significant ventilatory limitation (5), criteria 1, 2 and 4 may not be achieved despite maximal patient effort. Severe hypoxemia/gas exchange impairment, chest pain, ischemic ECG changes, and decreases in heart rate and blood pressure can occur during submaximal exercise and are not evidence of maximal effort [1], but may be very informative in the interpretation of test results.

## 13. Evaluation of Peak Oxygen Consumption

As ${\dot{V}}_{{\text{O}}_{\text{2peak}}}/{\dot{V}}_{{\text{O}}_{\text{2max}}}$  is affected by age and sex, conditioning status, and the presence of diseases or medications that can influence its components, accurate interpretation of exercise data requires reference values that are appropriate for each patient (see [1] for a comprehensive list of reference formulas). As with any criteria, the determination of low/abnormal ${\dot{V}}_{{\text{O}}_{\text{2max}}}/{\dot{V}}_{{\text{O}}_{2\text{peak}}}$ is somewhat arbitrary. The American Thoracic Society/American College of Chest Physicians statement on cardiopulmonary exercise testing defines a ${\dot{V}}_{{\text{O}}_{\text{2max}}}/{\dot{V}}_{{\text{O}}_{\text{2peak}}}\leq 84\%$  of predicted as abnormal [1]. When examining long-term survival, subjects with an absolute peak exercise capacity of >8 metabolic equivalents (METS) regardless of age, have improved survival as compared to subjects with a peak workload of 5–8 METS, or below 5 METS [100]. When exercise capacity is expressed as a % of predicted, subjects who attain a ${\dot{V}}_{{\text{O}}_{\text{2max}}}$  of 75%–100% of predicted have lower survival than those who reach ${\dot{V}}_{{\text{O}}_{\text{2max}}}>100\%$  of predicted, and survival is correspondingly lower for those with a ${\dot{V}}_{{\text{O}}_{\text{2max}}}$  50 to 74% and those with a ${\dot{V}}_{{\text{O}}_{\text{2max}}}<50\%$ of predicted, respectively [100]. These findings indicate that a ${\dot{V}}_{{\text{O}}_{\text{2max}}}$  below age-predicted, but still within typical values (i.e., 75%–100% of predicted), is associated with increased mortality and is therefore clinically important. 

${\dot{V}}_{{\text{O}}_{2\text{peak}}}/{\dot{V}}_{{\text{O}}_{\text{2max}}}$  is highly dependent on chronic physical fitness/exercise history and can be increased with exercise training and conversely reduced with inactivity. This is noteworthy when evaluating a previously athletic individual, as in these individuals a ${\dot{V}}_{{\text{O}}_{\text{2max}}}$  of ~100% of predicted may represent a substantial reduction in previous functional ability. The next section will now review how to determine whether the exercise intolerance can be explained by a pulmonary or cardiovascular limitation to exercise and whether this limitation is physiological (i.e., normal) or pathological.

## 14. Determining Exercise Limitation

**Figure 1:**
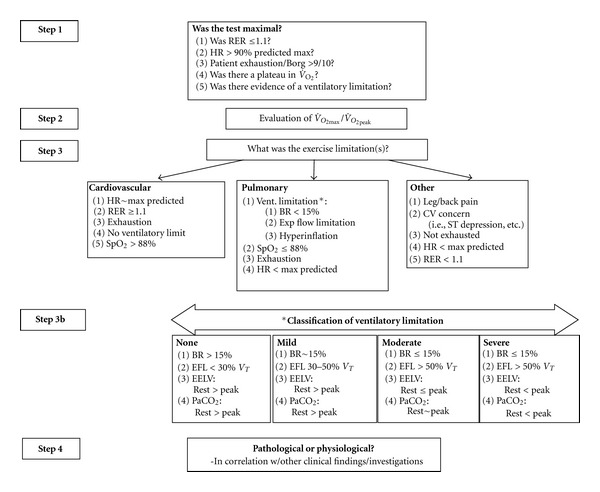
Interpretation algorithm for cardiopulmonary exercise testing. This figure provides an outline of a CPET interpretation strategy and suggested classification of ventilatory limitation based on previous work [1, 48, 53, 57, 58, 60, 70, 74]. Importantly, the data obtained from a CPET test should not be interpreted in isolation, but rather results should be integrated with other clinical findings/investigations. RER: respiratory exchange ratio, ${\dot{V}}_{{\text{O}}_{2}}\text{:}$ oxygen consumption, HR: heart rate, SpO_2_: arterial saturation, BR: breathing reserve, CV: cardiovascular, EFL: expiratory flow limitation, *V*_*T*_: tidal volume, EELV: end-expiratory lung volume, PaCO_2_: arterial PCO_2_.

Importantly, the data obtained from a CPET test should not be interpreted in isolation. Rather, the interpretation should be an integration of CPET results with other clinical findings/investigations. In addition to the data directly obtained from the CPET, feedback from the patient, including reason for exercise termination, can be useful in evaluating exercise limitation.  Figure 1 provides a guideline for CPET interpretation and classification based on previous work [48, 53, 57, 58, 60, 70, 74].

As detailed previously, ${\dot{V}}_{{\text{O}}_{\text{2max}}}$  is determined by the Fick equation. Increases in cardiac output/blood flow result in increased ${\dot{V}}_{{\text{O}}_{\text{2max}}}$, indicating that the normal person has a cardiovascular limitation to exercise. These subjects would surpass their ventilatory threshold, and therefore the RER would be expected to be >1.1, while HR should approach age-predicted maximum. In these subjects EFL, increases in EELV, and significant gas exchange impairment would not develop with exercise. Subjects who, despite showing a normal pulmonary, cardiovascular and metabolic response to exercise, still have a low  *V*_O_2max__  would be classified as being deconditioned. In contrast, subjects showing ECG changes with exercise, an exaggerated BP response to exercise, a significant drop in BP or HR with exercise, exaggerated ${\dot{V}}_{E}/{\dot{V}}_{{\text{CO}}_{2}}$ response with hyperventilation, and a very low ${\dot{V}}_{{\text{O}}_{\text{2max}}}$  would be suggestive of a pathological cardiovascular limitation to exercise. Thus, a cardiovascular limitation to exercise is the interpretation of default; that is, in the absence of any abnormal/pathological response, subjects are limited by their cardiovascular system.

When ventilatory demand is excessive or ventilatory capacity is reduced, a ventilatory limitation to exercise can develop. Ventilatory reserve is related to ventilatory demand, and ventilatory capacity [46, 48]; however because of the difficulties in determining MVV and the lack of information provided about the mechanism of ventilatory constraint, ventilatory reserve in isolation is a more rudimentary evaluation of ventilatory limitation, and determination of EFL and IC is preferable. As mentioned previously, EFL determination also has its limitations, and failure to account for variables such as thoracic gas compression and exercise-induced bronchodilation/bronchoconstriction will result in an overestimation of EFL [64]. Since an EFL < 25% of *V*_*T*_ can occur at maximal exercise in normal subjects [48, 49, 59, 60], it is unlikely that this amount of EFL should be considered abnormal and clinically significant. The development of EFL for >40%–50%  *V*_*T*_  is abnormal and can result in an increase in EELV [48, 53, 57, 58]. As EFL contributes to work of breathing and functional impairment of inspiratory muscle strength [55, 56], significant EFL by itself would contribute to perceived dyspnea and exercise intolerance. The development of EFL with a decrease in IC would represent a more severe respiratory limitation and also result in a plateau in tidal volume expansion and potentiated dyspnea [60, 74]. In the most severe cases, hypercapnea and hypoxemia would develop, as ventilation is insufficient to meet metabolic demand. In many cases, the ventilatory limitation to exercise is so severe that the patient does not reach their ventilatory threshold (i.e., an RER < 1.0 at peak) or age-predicted maximum heart rate. Some subjects demonstrate a reduction in IC with exercise despite normal lung function and no evidence of EFL or any other mechanical limitation. In these situations, behavioral conditions such as anxiety should be considered. See Figure 1 for a suggested classification of ventilatory limitation based on previous work [48, 53, 57, 58, 60, 70, 74].

The pulmonary system can further contribute to exercise intolerance by failing to maintain adequate arterial oxygenation. Previous guidelines indicate a fall in SaO_2_ of ≥4%, SaO_2_ ≤ 88% or PaO_2_ ≤ 55 mmHg is considered clinically significant [1]. As mentioned, SaO_2_/SpO_2_ evaluated in isolation does not allow for determination of the underlying mechanism for hypoxemia (i.e., hypoventilation versus gas exchange impairment versus lactic acidosis/hyperthermia). 

Poor ventilatory efficiency (i.e., high ${\dot{V}}_{E}/{\dot{V}}_{{\text{CO}}_{2}}$) can be characteristic of various cardiovascular and pulmonary diseases. Importantly, an abnormal ${\dot{V}}_{E}/{\dot{V}}_{{\text{CO}}_{2}}$ response may be a signal to obtain arterial blood gases during exercise so that PaCO_2_ and dead space ventilation can be directly determined [1]. A high ${\dot{V}}_{E}/{\dot{V}}_{{\text{CO}}_{2}}$ ratio in isolation may contribute to dyspnea but is not likely to contribute to exercise intolerance by itself. However, with an exaggerated ventilatory response to exercise EFL and an increase in EELV that may develop, and these components would contribute to exercise intolerance. 

Other patients may terminate a CPET because of alternate issues such as back pain and knee pain. In addition, the testing staff may terminate the exercise because of safety concerns (ECG changes, altered BP response, etc.). In these situations, the test would be terminated because of a noncardiopulmonary limitation, and it is unlikely that the patient would have reached maximal patient effort. 

As a final step, the clinician should determine whether the limitation to exercise is physiological (i.e., normal) or pathological and needing further followup. By way of example, a subject with a low ${\dot{V}}_{{\text{O}}_{2\text{peak}}}$, but otherwise normal test, would have a physiological cardiovascular limitation to exercise whereby the low ${\dot{V}}_{{\text{O}}_{2\text{peak}}}$ is explained by deconditioning. A subject with a similar ${\dot{V}}_{{\text{O}}_{2\text{peak}}}$, but showing abnormal ECG or BP responses, would have a pathological cardiovascular limitation requiring further followup. A COPD patient who has a low ${\dot{V}}_{{\text{O}}_{2\text{peak}}}$, but otherwise normal test (including a normal ventilatory response to exercise), would have a physiological cardiovascular limitation to exercise whereby the low ${\dot{V}}_{{\text{O}}_{2\text{peak}}}$ is explained by deconditioning. While in contrast, a COPD patient who has a low ${\dot{V}}_{{\text{O}}_{2\text{peak}}}$ but substantial EFL and hyperinflation would have a pathological respiratory limitation to exercise. Respiratory limitations to exercise are typically pathological, except in the case of an athlete with superior cardiovascular function and normal lung function [28]. These athletes can demonstrate EFL, increased EELV and gas exchange impairment; however, this is an example of the cardiovascular system outgrowing the lungs, and not pulmonary pathology [28]. Of note, patients may demonstrate evidence of both a cardiovascular and pulmonary limitation to exercise.

## 15. Summary

As reviewed in this paper, exercise represents a significant stress to the cardiopulmonary system. With exercise, oxygen delivery and local muscle O_2_ extraction must increase appropriately to meet metabolic demand. Ventilation must similarly increase to compensate for the increased CO_2_ production and maintain alveolar ventilation, while diffusion capacity must also be augmented to maintain arterial PO_2_. The normal subject has a breathing reserve even at maximal exercise, and therefore expiratory flow limitation and/or hyperinflation should not occur with exercise. In addition, healthy subjects maintain oxygenation up to peak exercise because of an appropriate increase in diffusion capacity. The failure to have an appropriate cardiovascular, ventilatory, or gas exchange response to exercise can result in greater exertional dyspnea and/or exercise tolerance. As outlined in the paper, examining the cardiopulmonary responses to a CPET can provide additional clinical data that is not available through resting tests of lung and cardiac function and can help clinicians determine mechanism(s) for exercise intolerance and/or dyspnea.

## Figures and Tables

**Table 1:** Indications for cardiopulmonary exercise testing.

Assessment of unexplained dyspnea	
Evaluation of disease severity	
Development of an exercise prescription for pulmonary	
rehabilitation	
Identification of gas exchange abnormalities	
Preoperative assessment:	
Lung cancer surgery	
Lung volume reduction surgery	
Heart or lung transplantation	
Evaluation for lung/heart transplantation	
Objective evaluation of exercise capacity	

**Table 2:** Contraindications for cardiopulmonary exercise testing.

Acute myocardial infarction
Unstable angina
Unstable arrhythmias
Syncope
Symptomatic severe aortic stenosis
Any acute pulmonary symptom
Any acute infectious process
Inability to comply with testing procedures

## Abbreviations

Alveolar PO_2_: PAO_2_
Alveolar ventilation: ${\dot{V}}_{A}$
Arterial O_2_ content: CaO_2_
Arterial PO_2_: PaO_2_
Arterial saturation: SaO_2_
Arterial saturation by pulse oximetry: SpO_2_
Cardiopulmonary exercise test: CPET
CO_2_ production: ${\dot{V}}_{{\text{CO}}_{2}}$
Diffusion capacity for carbon monoxide: DLCO
Diffusion capacity for O_2_: DLO_2_
End-tidal CO_2_: PETCO_2_
End-tidal O_2_: PETO_2_
Expiratory flow limitation: EFL
Expiratory lung volume: EELV
Expiratory time: *T*_*E*_
Heart rate: HR
Inspiratory capacity: IC
Inspiratory time: *T*_*I*_
Maximal oxygen consumption: ${\dot{V}}_{{\text{O}}_{\text{2max}}}$
Maximal voluntary ventilation: MVV
Metabolic equivalents: METS
Minute ventilation: ${\dot{V}}_{E}$
Mixed venous O_2_ content: CvO_2_
Peak minute ventilation: ${\dot{V}}_{E\max}$
Peak oxygen consumption: ${\dot{V}}_{{\text{O}}_{2\text{peak}}}$
Physiologic dead space ventilation: ${\dot{V}}_{D}$
Pulmonary arterial hypertension: PAH
Tidal volume: *V*_*T*_
Total breath cycle duration: *T*_TOT_
Total lung capacity: TLC
Ventilation/perfusion: ${\dot{V}}_{a}/\dot{Q}$.
